# Nonalcoholic fatty liver disease in relation to the remission and progression along the glycemic continuum

**DOI:** 10.1111/1753-0407.13314

**Published:** 2022-09-26

**Authors:** Zhuojun Xin, Jiaojiao Huang, Qiuyu Cao, Jialu Wang, Ruixin He, Tianzhichao Hou, Yi Ding, Jieli Lu, Min Xu, Tiange Wang, Zhiyun Zhao, Weiqing Wang, Guang Ning, Yufang Bi, Yu Xu, Mian Li

**Affiliations:** ^1^ Department of Endocrine and Metabolic Diseases, Shanghai Institute of Endocrine and Metabolic Diseases Ruijin Hospital, Shanghai Jiao Tong University School of Medicine Shanghai China; ^2^ Shanghai National Clinical Research Center for Metabolic Diseases, Key Laboratory for Endocrine and Metabolic Diseases of the National Health Commission of the PR China, Shanghai Key Laboratory for Endocrine Tumor, State Key Laboratory of Medical Genomics Ruijin Hospital, Shanghai Jiao Tong University School of Medicine Shanghai China

**Keywords:** epidemiology, glycemic profile, nonalcoholic fatty liver disease, remission, type 2 diabetes, 流行病学, 血糖谱, 非酒精性脂肪性肝病, 缓解, 2型糖尿病

## Abstract

**Background:**

The study aimed to explore the associations of nonalcoholic fatty liver disease (NAFLD) with the remission and progression along the glycemic continuum.

**Methods:**

This prospective cohort study was performed among the general population in 2010–2015. NAFLD was defined as ultrasound‐detected hepatic steatosis with absence of excessive alcohol consumption and other hepatic diseases. Remission of type 2 diabetes referred to glycated hemoglobin <6.5% without hypoglycemic agents for ≥3 months. Prediabetes remission referred to normalization of blood glucose. Multivariable logistic analysis was applied to identify the risk of glycemic metabolic transition.

**Results:**

During a median follow‐up of 4.3 years, participants with NAFLD had a significantly higher risk of progressing from normal glucose tolerance to diabetes (3.36 [1.60–7.07]) and lower likelihood of diabetes remission (0.48 [0.30–0.78]). Associations in participants with overweight or obesity and higher probability of hepatic fibrosis remained consistent. Results related to the effect of NAFLD on the specific glucose parameters were generally in line with the changes of glycemic status. NAFLD improvement decreased the risk of prediabetes progressing to diabetes (0.50 [0.32–0.80]) and increased the probability of prediabetes remission (2.67 [1.49–4.79]). NAFLD tended to show the most significant association with glycemic progression and decreased the likelihood in remission of prediabetes and diabetes.

**Conclusions:**

Presence of NAFLD increased risk of glycemic progression and decreased likelihood of remission. NAFLD improvement mitigated glycemic deterioration, whereas NAFLD progression impeded the chance of remission. The results emphasized joint management of NAFLD and diabetes and further focused on liver‐specific subgroups of diabetes to tailor early intervention.

## Graphical abstract

**Highlights**

- To what extent NAFLD is associated with glycemic remission among the general population remains unclear.
- The study elaborated that presence of NAFLD and fibrosis status decreased the chance of glycemic remission whereas NAFLD improvement mitigated glycemic deterioration, emphasizing joint management to liver‐focused subgroups of diabetes.

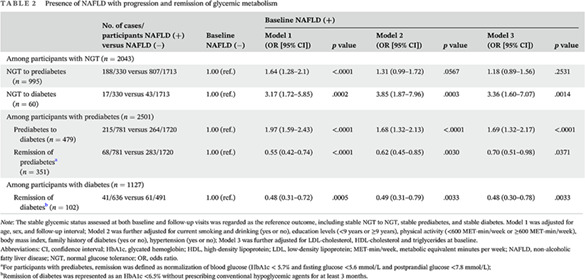

## 摘要

**背景:**

本研究旨在探讨非酒精性脂肪性肝病(NAFLD)与糖尿病进展的关系。

**方法:**

这项前瞻性队列研究于2010‐2015年在普通人群中进行。NAFLD被定义为超声检测到的肝脏脂肪变性，没有过量饮酒和其他肝病。2型糖尿病的缓解是指在≥3个月内未服用降糖药的患者糖化血红蛋白低于6.5%。糖尿病前期缓解是指血糖正常化。多变量Logistic分析用于识别血糖代谢转换的风险。

**结果:**

在平均4.3年的随访期间，NAFLD的参与者从正常糖耐量(NGT)进展到糖尿病的风险较高[3.36(1.60‐7.07)]，而糖尿病缓解的可能性较低[0.48(0.30‐0.78)]。超重或肥胖与较高的肝纤维化概率之间的关联仍然是一致的。NAFLD对特定血糖参数的影响与血糖状态的变化基本一致。NAFLD的改善降低了糖尿病前期进展为糖尿病的风险[0.50(0.32‐0.80)]，并增加了糖尿病前期缓解的可能性[2.67(1.49‐4.79)]。NAFLD倾向于表现出与血糖进展最显著的相关性，并降低了糖尿病前期和糖尿病缓解的可能性。

**结论:**

NAFLD的存在增加了高血糖进展的风险，降低了缓解的可能性。NAFLD的改善可缓解血糖恶化，而NAFLD的进展则阻碍缓解的机会。应该强调NAFLD和糖尿病的联合管理，并进一步关注糖尿病的肝脏特异性亚组，以量身定制早期干预。

## 1 INTRODUCTION

Type 2 diabetes (T2D), now has reached epidemic proportions, affecting 10.5% adults worldwide and 10.6% in China, imposing great burden not only on individuals but also society.^1^ T2D has been perceived as progressive and irreversible condition for a long time.^2^, ^3^ Recently, accumulating evidence based on intensive lifestyle intervention studies has proved the attainability of T2D remission, such as the Diabetes Remission Clinical Trial (DiRECT) study, which achieved 46% remission among short‐duration T2D, apart from bariatric surgery and intensive insulin therapy.^4^, ^5^, ^6^ Nevertheless, participants have inconsistent response to intensive lifestyle intervention, indicating that there were individual differences in the achievement of T2D remission. Results from the DiRECT study demonstrated that remission of T2D caused by sufficient weight loss was largely depended on the decrease in hepatic fat content, implying the critical role of hepatic fat accumulation in T2D remission.^7^

In 2021, the American Diabetes Association (ADA) proposed the definition and interpretation of remission in T2D, as well as an urgent need to detect potential predictors for remission.^8^ Nevertheless, outside of the context of clinical trials, data are still limited regarding the characteristics of people who were in remission of T2D in normal care. Recently, a retrospective cohort study has detected the inverse association of fatty liver and T2D remission among 2567 diabetic Japanese over a 2‐year follow‐up period, for the first time indicating a potential effect of nonalcoholic fatty liver disease (NAFLD) on T2D remission in a real world situation.^9^ Actually, on account of the prevalence and long‐term risk related to prediabetes, prediabetes and T2D were recognized as a continuum of glucose intolerance, which raised the importance to recognize the potential markers of progression and remission on the unidirectional road of glycemic metabolism.

Hence, based on a prospective cohort study among the Chinese general population, the current study aimed to further fill the following gaps: (1) associations of NAFLD status with the remission and progression across normal glucose tolerance (NGT), prediabetes, and T2D and the change of the glycemic metrics along the glycemic continuum; and (2) associations of changes of NAFLD status and degrees of fibrosis status with glycemic metabolic transition.

## 2 METHODS

### 2.1 Study design and population

**FIGURE 1:**
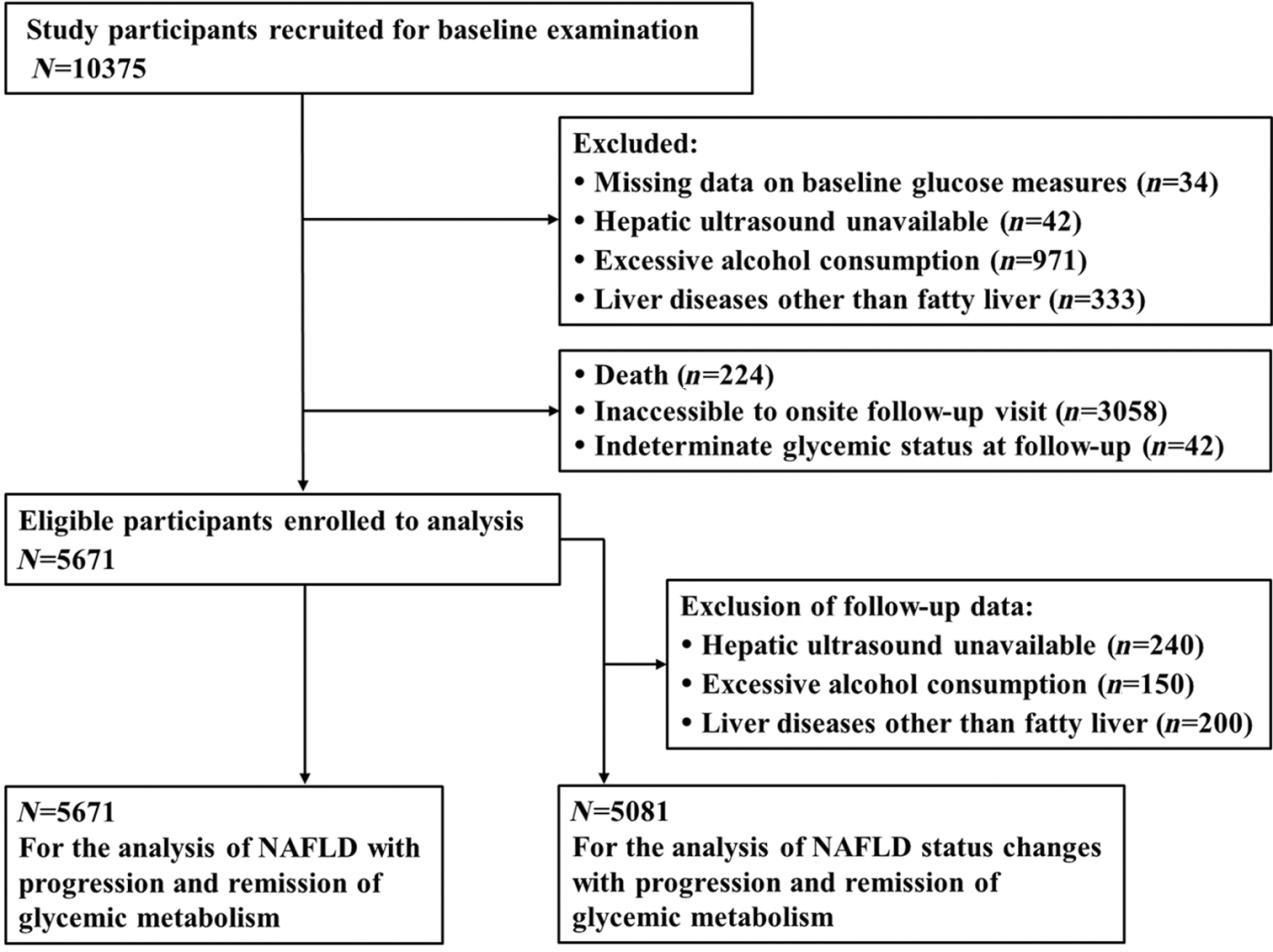
Study population flow diagram. NAFLD, non‐alcoholic fatty liver disease.

The study was launched among a community‐based population in Jiading District of Shanghai, China between March and August 2010. The protocol has been described in detail elsewhere.^10^ Briefly, a total of 10 375 registered residents aged ≥40 years were recruited and completed a baseline health examination, including a comprehensive standard questionnaire and clinical measurements. After a follow‐up of up to 5 years, the enrolled participants were reinvited for a follow‐up visit during August 2014 and May 2015. For the current analysis, participants with indeterminate information on identification of NAFLD and glycemic status were excluded: (1) missing data on baseline glucose parameters (*n* = 34); (2) incompletion of hepatic ultrasound (*n* = 42); (3) excessive alcohol consumption (*n* = 971) and liver diseases other than fatty liver (*n* = 333); (4) registered for death during the follow‐up (*n* = 224); (5) inaccessible to onsite follow‐up visit (*n* = 3058); and (6) indeterminate to identify glycemic status at follow‐up (*n* = 42). To detect the NAFLD status changes between two visits, missing data on the definition of follow‐up NAFLD were further excluded (*n* = 590). Finally, 5671 participants were included in the analysis of associations of NAFLD with progression and remission of glycemic metabolism, and 5081 were analyzed for NAFLD status changes, respectively. Detailed selection procedure was presented in Figure 1. The study protocol was endorsed by the Institutional Review Board of Rui‐Jin Hospital. Detailed written informed consent was obtained from each participant.

### 2.2 Measurements

Data on demographic profile, educational attainment, lifestyle factors (including cigarette smoking, alcoholic consumption, and physical activity) and medical history were collected at baseline and follow‐up visits by standard questionnaires face to face. Self‐reported illness and medication will be further confirmed by the carry‐on medical records. Physical activity was assessed by the short form of the Global Physical Activity Questionnaire and was classified into two groups: ≥600 metabolic equivalent minutes per week (MET‐min/week) or not^11^ Body weight, height, and blood pressure were measured by trained staff on the basis of a standard protocol.^12^ Body mass index (BMI) was calculated as weight in kilograms divided by height in meters squared (kg/m^2^).

All participants underwent an oral glucose tolerance test after an overnight fasting (>10 h). Fasting and 2‐hour postprandial plasma glucose were measured by the glucose oxidase method on an automated analyzer (Modular Analytics P800; Roche). Glycated hemoglobin (HbA1c) was determined by high‐performance liquid chromatography using the VARIANT II Hemoglobin Testing System (Bio‐Rad Laboratories). Fasting serum insulin and biochemical parameters, including total cholesterol, triglycerides, low‐density lipoprotein (LDL) cholesterol, and high‐density lipoprotein (HDL) cholesterol, as well as liver enzymes (including alanine aminotransferase, aspartate aminotransferase, and γ‐glutamyl‐transferase) were measured on auto‐analyzers (Modular E170; Roche).

### 2.3 Ultrasound‐based definition of NAFLD

Liver ultrasound was performed separately by two clinical sonographers, using a high‐resolution B‐mode tomographic ultrasonic system (Esaote Biomedica SpA, Italy) with a 3.5‐MHz probe. Fatty liver was detected by the presence of ≥2 of following three imaging findings: (1) diffusely increased echogenicity of the liver relative to the kidney, (2) ultrasound beam attenuation, or (3) poor visualization of intrahepatic structures. A third sonographer, who was blinded to the study, was required if there was contradictory between the former two diagnosis of fatty liver. Definition of NAFLD was based on ultrasound‐detected fatty liver with absence of excessive alcohol consumption and other hepatic diseases.^13^, ^14^

To further clarify associations of NAFLD status changes and glycemic metabolism, the presence of NAFLD was evaluated both at baseline and follow‐up visits. Study participants were classified into four groups according to the two visits’ presence status of NAFLD: (1) stable non‐NAFLD; (2) new‐onset NAFLD; (3) stable NAFLD; and (4) NAFLD improvement, respectively.

The probability of hepatic fibrosis was assessed by the invasive index of fibrosis‐4 (FIB‐4) and was calculated as follows: FIB‐4 = age (years) × aspartate aminotransferase (U/L)/(platelet count [×10^9^/L] × alanine aminotransferase [U/L]^1/2^). Higher probability of hepatic fibrosis was identified as FIB‐4 ≥ 1.3.^15^

### 2.4 Definition of glycemic status

T2D was diagnosis as HbA1c ≥ 6.5% or fasting plasma glucose ≥126 mg/dl or 2‐h postprandial plasma glucose ≥200 mg/dl, or with a previous diagnosis of diabetes, according to the ADA criteria.^16^ Prediabetes was defined as HbA1c 5.7%–6.4% or fasting plasma glucose 100–125 mg/dl, or 2‐h postprandial plasma glucose 140–199 mg/dl. The index of homeostasis model assessment of insulin resistance (HOMA‐IR) was calculated as fasting serum insulin (μIU/ml) × fasting plasma glucose (mmol/L)/22.5. HOMA‐IR ≥ 2.5 was defined as insulin resistance.^17^

Glycemic progression referred to (1) NGT at baseline progressing to new‐onset diabetes and new‐onset prediabetes at follow‐up, and (2) baseline prediabetes status progressing to diabetes at follow‐up, respectively.

Glycemic remission contained remission of type 2 diabetes and prediabetes at follow‐up. According to ADA consensus updated in 2021, remission of T2D referred to HbA1c below 6.5% on the premise of not prescribing conventional hypoglycemic agents for at least 3 months.^8^ Prediabetes remission referred to normalization of blood glucose (HbA1c < 5.7% and fasting plasma glucose <100 mg/dl and 2‐hour postprandial plasma glucose <140 mg/dl). The routine medication history of insulin or hypoglycemic agents was first obtained by the standard question “Have you ever injected insulin or taken any hypoglycemic agents in the past 3 months?” and then confirmed according to the medical records.

### 2.5 Statistical analysis

Continuous variables were presented by means ± SD or medians (interquartile ranges), and categorical variables were expressed in numbers (percentages). Baseline characteristics were summarized according to the combination of the baseline status of NAFLD and glycemic metabolism.

Multivariable logistic model was fitted to investigate associations of NAFLD status with progression and remission of glycemic metabolism. Study population was divided into three groups according to baseline glycemic status: NGT, prediabetes, and diabetes. To specify the progression and remission of glycemic metabolism, stable glycemic status evaluated at baseline and follow‐up visits, including stable NGT, stable prediabetes, and stable diabetes were regarded as reference outcomes in respective groups. Odds ratio (OR) and 95% confidence interval (CI) were calculated after potential adjustments for age, sex, follow‐up interval (model 1), current smoking and drinking status (yes or no), education levels (<9 years or ≥9 years), physical activity (<600 MET‐min/week or ≥ 600 MET‐min/week), BMI, family history of diabetes (yes or no), hypertension (yes or no) (model 2), LDL‐cholesterol, HDL‐cholesterol, and triglycerides (model 3). Changes of specific glucose parameters (HbA1c, fasting glucose, 2‐h glucose, and status of insulin resistance) were also analyzed. Categories of normal, impaired/elevated, and diabetic status were corresponding to the diagnostic ranges of NGT, prediabetes, and diabetes, respectively. For the analysis of NAFLD status changes and glycemic metabolism, the presence of NAFLD was evaluated not only at baseline but also follow‐up visit. Associations between NAFLD changes and transition of glycemic metabolism were further explored.

Statistical analysis was performed on SAS 9.2 and a two‐tailed *p* value <.05 was of statistical significance.

## 3 RESULTS

### 3.1 General characteristics

Baseline characteristics of the included 5671 participants in the associations of NAFLD with progression and remission of glycemic metabolism were summarized in Table 1 according to the combination of the baseline status of NAFLD and glycemic metabolism. In the analyzed population, 1747 (30.8%) were NAFLD and 3924 (69.2%) were non‐NAFLD. Mean ages in the two groups were 58.2 (8.3) and 58.0 (9.0), respectively. In general, NAFLD participants had a worse performance in the baseline metabolic characteristics compared with non‐NAFLD counterparts (all *p* < .05). Combined with the baseline glycemic status, the prevalence of prediabetes and diabetes was 44.7% and 36.4% for NAFLD group and 43.8% and 12.5% for non‐NAFLD group, respectively. Compared with participants in both NGT subgroups, those with prediabetes or diabetes tended to be older, with lower educational levels and higher proportion of diabetes family history (all *p* for trend <.05). Concerning to cardiometabolic health indicators, participants with prediabetes and diabetes had higher levels of systolic blood pressure, adipose measurements (BMI and waist circumference), glucose parameters (including fasting and 2‐hour postprandial glucose, HbA1c, and HOMA‐IR), lipid profiles (total cholesterol, triglycerides, and LDL‐cholesterol), and liver enzymes, along with the deterioration of glucose status (all *p* for trend <.05).

**TABLE 1:** Baseline characteristics of study population according to the presence of NAFLD and glycemic status at baseline

	NAFLD	Non‐NAFLD	NAFLD				Non‐NAFLD			
			NGT	Prediabetes	Diabetes	*p* for trend	NGT	Prediabetes	Diabetes	*p* for trend
No. of participants, *n* (%)	1747 (30.8)	3924 (69.2)	330 (18.9)	781 (44.7)	636 (36.4)	‐	1713 (43.7)	1720 (43.8)	491 (12.5)	‐
Age, year	58.2 ± 8.3	58.0 ± 9.0	55.3 ± 7.7	58.1 ± 8.1	59.8 ± 8.4	<.0001	55.7 ± 8.7	59.2 ± 8.8	61.9 ± 8.7	<.0001
Male, *n* (%)	533 (30.5)	1143 (29.13)	112 (33.9)	210 (26.9)	211 (33.2)	.6850	488 (28.5)	467 (27.2)	188 (38.3)	.0039
High school or above, *n* (%)	346 (19.8)	776 (19.8)	85 (25.8)	145 (18.6)	116 (18.2)	.0139	388 (22.7)	288 (16.7)	100 (20.4)	.0064
Current smoking, *n* (%)	309 (17.7)	655 (16.7)	70 (21.2)	118 (15.1)	121 (19.0)	.7864	311 (18.2)	260 (15.1)	84 (17.1)	.1482
Current drinking, *n* (%)	223 (12.8)	462 (11.8)	52 (15.8)	90 (11.5)	81 (12.7)	.3182	208 (12.1)	184 (10.7)	70 (14.3)	.6695
Family history of diabetes, *n* (%)	268 (15.4)	383 (9.8)*	36 (10.9)	93 (11.9)	139 (21.9)	<.0001	128 (7.5)	140 (8.2)	115 (23.5)	<.0001
Physical activity ≥ 600 MET‐min/week, *n* (%)	1233 (70.6)	2828 (72.1)	241 (73.0)	537 (68.8)	455 (71.5)	.8862	1225 (71.5)	1241 (72.2)	362 (73.7)	.3559
Body mass index (kg/m^2^)	27.5 ± 3.0	24.2 ± 2.8*	27.3 ± 2.8	27.5 ± 2.9	27.7 ± 3.3	.0715	23.9 ± 2.7	24.3 ± 2.8	24.6 ± 3.1	<.0001
Waist circumference (cm)	88.6 ± 7.8	79.4 ± 7.7*	88.1 ± 7.1	88.0 ± 7.6	89.8 ± 8.3	.0002	78.4 ± 7.6	79.7 ± 7.6	81.9 ± 7.7	<.0001
Systolic BP (mmHg)	146.6 ± 19.1	139.0 ± 19.9*	140.5 ± 17.1	146.4 ± 18.8	150.0 ± 19.7	<.0001	134.9 ± 19.6	140.8 ± 19.4	147.5 ± 19.2	<.0001
Diastolic BP (mmHg)	85.6 ± 10.3	81.4 ± 10.0*	85.0 ± 9.7	86.0 ± 10.3	85.4 ± 10.6	.8525	80.8 ± 10.1	82.0 ± 9.8	81.5 ± 10.3	.0138
Fasting glucose (mg/dl)	109.8 ± 35.6	95.8 ± 21.2*	88.3 ± 7.2	96.7 ± 10.7	136.9 ± 46.0	<.0001	86.9 ± 6.9	94.8 ± 10.3	129.9 ± 40.1	<.0001
Postprandial glucose (mg/dL)	185.5 ± 92.8	134.2 ± 65.0*	111.2 ± 20.0	142.4 ± 30.3	277.2 ± 93.8	<.0001	103.3 ± 20.1	128.9 ± 31.5	261.9 ± 96.6	<.0001
HbA1c (%)	6.2 ± 1.2	5.7 ± 0.7*	5.4 ± 0.2	5.8 ± 0.3	7.1 ± 1.5	<.0001	5.3 ± 0.2	5.7 ± 0.3	6.8 ± 1.5	<.0001
HOMA‐IR	2.6 (1.8–3.8)	1.4 (1.0–1.9)*	2.0 (1.5–2.7)	2.4 (1.8–3.3)	3.5 (2.4–5.2)	<.0001	1.2 (0.9–1.7)	1.4 (1.0–2.0)	2.0 (1.3–3.1)	<.0001
Total cholesterol (mg/dl)	213.0 ± 42.7	205.4 ± 37.7*	204.4 ± 36.9	215.9 ± 46.1	214.1 ± 40.6	.0060	200.6 ± 37.3	209.2 ± 36.9	208.9 ± 40.2	<.0001
Triglycerides (mg/dl)	164.6 (120.4–229.2)	109.7 (80.5–149.6)*	158.0 (113.3–212.4)	164.6 (120.4–228.3)	169.5 (122.6–231.4)	.0113	102.7 (77.0–140.7)	111.5 (82.3–155.8)	122.1 (89.4–158.4)	<.0001
LDL‐cholesterol (mg/dl)	128.0 ± 35.1	123.1 ± 32.6*	121.7 ± 31.9	130.2 ± 36.2	128.6 ± 35.0	.0209	119.0 ± 32.3	126.0 ± 31.6	127.6 ± 35.3	<.0001
HDL‐cholesterol (mg/dl)	46.7 ± 10.4	53.2 ± 12.4*	45.6 ± 10.5	47.1 ± 10.2	46.8 ± 10.6	.1870	53.5 ± 12.1	53.5 ± 12.8	51.1 ± 12.0	.0028
Alanine aminotransferase (IU)	23.2 (17.4–33.4)	16.5 (12.9–21.8)*	20.3 (15.8–27.6)	22.9 (17.4–32.1)	25.0 (18.1–38.8)	<.0001	16.0 (12.6–21.1)	16.7 (13.2–21.6)	17.6 (13.4–23.8)	<.0001
Aspartate aminotransferase (IU)	22.4 (19.0–27.4)	21.1 (18.1–24.6)*	21.1 (17.9–25.3)	22.6 (19.2–27.4)	23.0 (19.4–29.8)	<.0001	20.9 (18.0–24.4)	21.5 (18.5–24.9)	20.2 (16.9–24.6)	.8902
γ‐glutamyl transferase (IU)	29.0 (10.0–44.0)	18.0 (13.0–26.0)*	23.0 (17.0–36.0)	28.0 (20.0–43.0)	32.0 (22.0–50.0)	<.0001	16.0 (12.0–24.0)	18.0 (14.0–26.0)	21.0 (16.0–31.0)	<.0001
Fibrosis‐4 ≥ 1.3, *n* (%)	791 (45.3)	2246 (57.2)*	143 (43.3)	345 (44.2)	303 (47.6)	.1537	940 (54.9)	1026 (59.7)	280 (57.0)	.0619

* *p* < .05 for baseline differences between the NAFLD and non‐NAFLD groups.

Abbreviations: BP, blood pressure; HbA1c, glycated hemoglobin; HDL, high‐density lipoprotein; HOMA‐IR, homeostasis model assessment of insulin resistance; LDL, low‐density lipoprotein; MET‐min/week, metabolic equivalent minutes per week; NAFLD, non‐alcoholic fatty liver disease; NGT, normal glucose tolerance.

### 3.2 Presence of NAFLD with progression and remission of glycemic metabolism

Table 2 exhibited the associations of NAFLD presence with progression and remission of glycemic metabolism. During a median follow‐up of 4.3 years, 57.0% (188/330) and 5.2% (17/330) NAFLD participants with NGT progressed to new‐onset prediabetes and diabetes, respectively. Meanwhile, 8.7% (68/781) and 6.4% (41/636) participants with NAFLD were in remission of prediabetes and diabetes, respectively.

**TABLE 2:** Presence of NAFLD with progression and remission of glycemic metabolism

	No. of cases/participants NAFLD (+) versus NAFLD (−)	Baseline NAFLD (−)	Baseline NAFLD (+)					
			Model 1 (OR [95% CI])	*p* value	Model 2 (OR [95% CI])	*p* value	Model 3 (OR [95% CI])	*p* value
Among participants with NGT (*n* = 2043)								
NGT to prediabetes (*n* = 995)	188/330 versus 807/1713	1.00 (ref.)	1.64 (1.28–2.1)	<.0001	1.31 (0.99–1.72)	.0567	1.18 (0.89–1.56)	.2531
NGT to diabetes (*n* = 60)	17/330 versus 43/1713	1.00 (ref.)	3.17 (1.72–5.85)	.0002	3.85 (1.87–7.96)	.0003	3.36 (1.60–7.07)	.0014
Among participants with prediabetes (*n* = 2501)								
Prediabetes to diabetes (*n* = 479)	215/781 versus 264/1720	1.00 (ref.)	1.97 (1.59–2.43)	<.0001	1.68 (1.32–2.13)	<.0001	1.69 (1.32–2.17)	<.0001
Remission of prediabetes^a^ (*n* = 351)	68/781 versus 283/1720	1.00 (ref.)	0.55 (0.42–0.74)	<.0001	0.62 (0.45–0.85)	.0030	0.70 (0.51–0.98)	.0371
Among participants with diabetes (*n* = 1127)								
Remission of diabetes^b^ (*n* = 102)	41/636 versus 61/491	1.00 (ref.)	0.48 (0.31–0.72)	.0005	0.49 (0.31–0.79)	.0033	0.48 (0.30–0.78)	.0033

*Note*: The stable glycemic status assessed at both baseline and follow‐up visits was regarded as the reference outcome, including stable NGT to NGT, stable prediabetes, and stable diabetes. Model 1 was adjusted for age, sex, and follow‐up interval; Model 2 was further adjusted for current smoking and drinking (yes or no), education levels (<9 years or ≥9 years), physical activity (<600 MET‐min/week or ≥600 MET‐min/week), body mass index, family history of diabetes (yes or no), hypertension (yes or no); Model 3 was further adjusted for LDL‐cholesterol, HDL‐cholesterol and triglycerides at baseline.

Abbreviations: CI, confidence interval; HbA1c, glycated hemoglobin; HDL, high‐density lipoprotein; LDL, low‐density lipoprotein; MET‐min/week, metabolic equivalent minutes per week; NAFLD, non‐alcoholic fatty liver disease; NGT, normal glucose tolerance; OR, odds ratio.

^a^ For participants with prediabetes, remission was defined as normalization of blood glucose (HbA1c < 5.7% and fasting glucose <5.6 mmol/L and postprandial glucose <7.8 mmol/L);

^b^ Remission of diabetes was represented as an HbA1c <6.5% without prescribing conventional hypoglycemic agents for at least 3 months.

Overall, the presence of NAFLD at baseline increased the risk of glycemic progression and decreased the probability of glycemic remission compared with non‐NAFLD counterparts. Specifically, after adjusting for all potential confounders, participants with NAFLD had a significantly higher risk of progressing from NGT to diabetes (OR 3.36; 95% CI 1.60–7.07) and a lower likelihood of diabetes remission (OR 0.48; 95% CI 0.30–0.78) compared with the non‐NAFLD group. Similar pattern of glycemic transition was additionally observed in participants with prediabetes (OR 1.69; 95% CI 1.32–2.17 for progressing from prediabetes to diabetes, and OR 0.70; 95% CI 0.51–0.98 for prediabetes remission). In the sensitivity analysis, fat‐free mass (FFM) assessed by bioelectrical impedance was further adjusted based on Model 3. Results related to the risk of progressing from NGT to diabetes (OR 3.24; 95% CI 1.43–7.32), from prediabetes to diabetes (OR 1.71; 95% CI 1.31–2.23) and remission of diabetes (OR 0.45; 95% CI 0.27–0.76), remained significant (Table S1).

**FIGURE 2:**
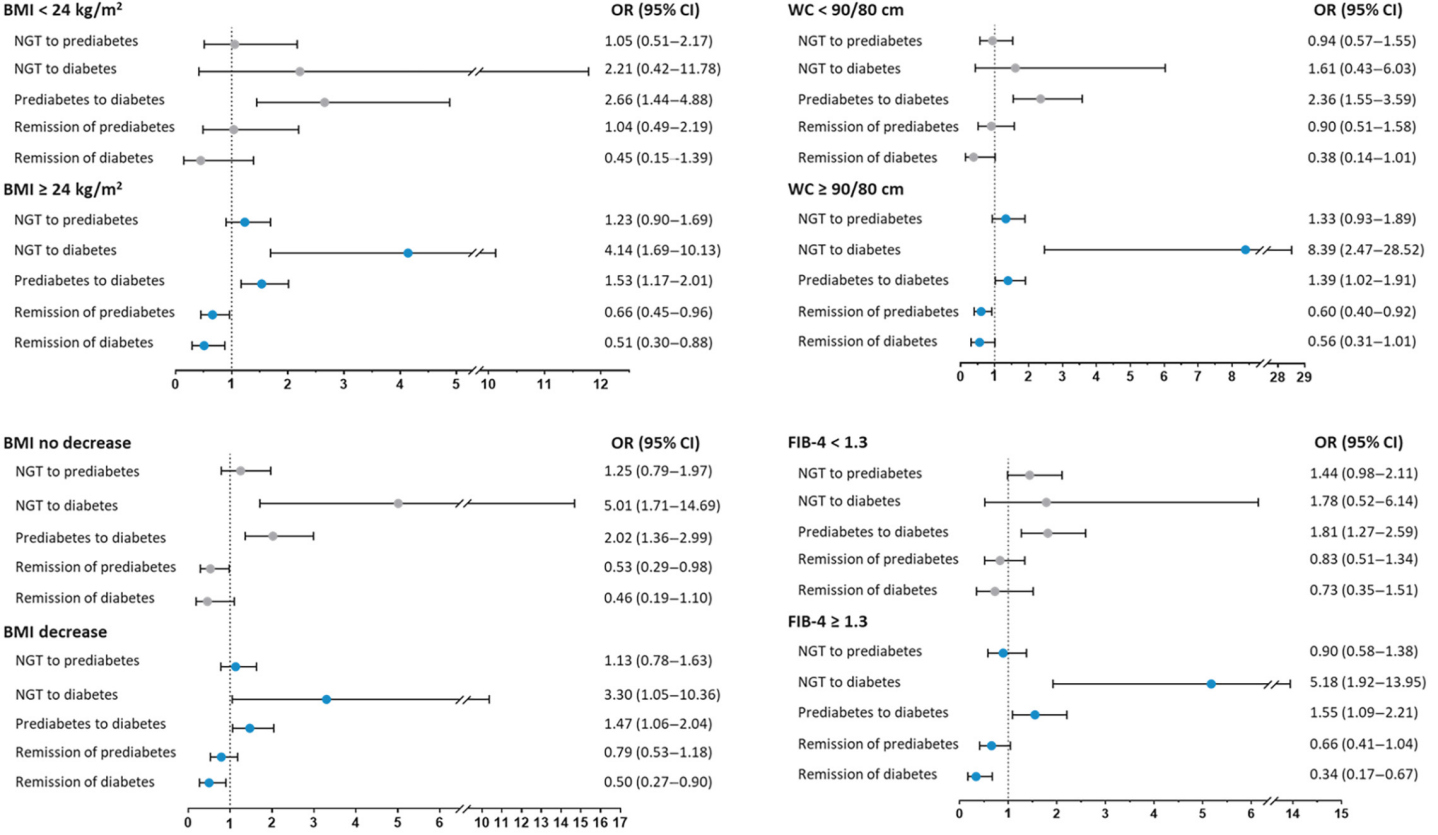
Stratified analyses according to BMI, BMI change, WC, and FIB‐4. The stable glycemic status at two visits of baseline and follow‐up, including stable NGT, stable prediabetes and stable diabetes were regarded as the reference outcome. Multivariable models were adjusted for age, sex, follow‐up interval, current smoking and drinking (yes or no), education levels (<9 years or ≥9 years), physical activity (<600 MET‐min/week or ≥ 600 MET‐min/week), BMI, family history of diabetes (yes or no), hypertension (yes or no), LDL‐cholesterol, HDL‐cholesterol, and triglycerides at baseline. BMI, body mass index; CI, confidence interval; FIB‐4, fibrosis‐4; HDL, high‐density lipoprotein; LDL, low‐density lipoprotein; MET‐min/week, metabolic equivalent minutes per week; NGT, normal glucose tolerance; OR, odds ratio; WC, waist circumference.

Stratified analyses were further conducted according to BMI, BMI change, waist circumference, and FIB‐4. As shown in Figure 2, NAFLD participants who had overweight or obesity (OR 4.14; 95% CI 1.69–10.13), central obesity (OR 8.39; 95% CI 2.47–28.52), or higher probability of hepatic fibrosis (OR 5.18; 95% CI 1.92–13.95) were associated with an elevated risk of progressing from NGT to diabetes and lower likelihood of diabetes remission (OR 0.51; 95% CI 0.30–0.88 among those with overweight or obesity; OR 0.34; 95% CI 0.17–0.67 among those with higher probability of hepatic fibrosis, respectively). Similar trend of glycemic transition was observed in participants with prediabetes. The level of BMI from baseline to the follow‐up visit tended to decrease (26.3 ± 3.5 vs. 25.8 ± 3.8 kg/m^2^) and the population was stratified into BMI decrease or not. The analysis showed that presence of NAFLD increased the risk of incident diabetes (OR 5.01; 95% CI 1.71–14.69) and impeded prediabetes remission (OR 0.50; 95% CI 0.27–0.90) even in participants without BMI decrease.

### 3.3 Association of NAFLD with changes of specific glucose parameters

In addition to elaborating the overall transition of glycemic status, we further investigated the association of NAFLD with changes of specific glucose parameters. Findings from Table 3 showed that NAFLD was involved in the whole process of glucose regulation, including HbA1c, fasting glucose, 2‐h glucose, and status of insulin resistance. With regard to HbA1c, an indicator of overall level of recent 2–3 months' glycemic status, its increased risk of progressing from elevated level to diabetes (OR 4.79; 95% CI 2.48–9.28) and decreased likelihood of regressing from diabetes to elevated or normal level (OR 0.25; 95% CI 0.08–0.77) were observed among participants with NAFLD. Significant similarities were found in the progression and remission of fasting glucose and 2‐h glucose parameters. As for the status of insulin resistance, NAFLD participants were more likely to get involved in the deterioration (OR 2.27; 95% CI 1.87–2.75) and less likely to improve to the normal status (OR 0.59; 95% CI 0.44–0.78). Results related to the effect of NAFLD on the glucose parameters were generally consistent with the changes of glycemic status presented in Table 2.

**TABLE 3:** Association of NAFLD with changes of specific glucose parameters

	No. of cases/participants NAFLD (+) versus NAFLD (−)	Baseline NAFLD (+)					
		Model 1	*p* value	Model 2	*p* value	Model 3	*p* value
		(OR [95% CI])		(OR [95% CI])		(OR [95% CI])	
HbA1c							
Stable normal (*n* = 2269)	415/1197 versus 1854/3332	1.00 (ref.)		1.00 (ref.)		1.00 (ref.)	
Normal to elevated (*n* = 293)	87/1197 versus 206/3332	1.80 (1.37–2.37)	<.0001	1.47 (1.07–2.02)	.0181	1.24 (0.89–1.73)	.2111
Normal to diabetic (*n* = 13)	7/1197 versus 6/3332	4.98 (1.65–15.00)	.0044	2.82 (0.74–10.82)	.1301	3.46 (0.72–16.62)	.1207
Stable elevated (*n* = 732)	213/1197 versus 519/3332	1.00 (ref.)		1.00 (ref.)		1.00 (ref.)	
Elevated to diabetic (*n* = 72)	50/1197 versus 22/3332	5.66 (3.33–9.62)	<.0001	5.38 (2.92–9.91)	<.0001	4.79 (2.48–9.28)	<.0001
Elevated to normal (*n* = 711)	164/1197 versus 547/3332	0.73 (0.58–0.93)	.0107	0.94 (0.72–1.23)	.6559	1.01 (0.76–1.34)	.9479
Stable diabetic (*n* = 413)	253/1197 versus 160/3332	1.00 (ref.)		1.00 (ref.)		1.00 (ref.)	
Diabetic to elevated/normal (*n* = 26)	8/1197 versus 18/3332	0.28 (0.12–0.67)	.0044	0.28 (0.10–0.74)	.0110	0.25 (0.08–0.77)	.0163
Fasting glucose							
Stable normal (*n* = 2062)	383/1204 versus 1679/3399	1.00 (ref.)		1.00 (ref.)		1.00 (ref.)	
Normal to impaired (*n* = 1398)	325/1204 versus 1073/3399	1.34 (1.14–1.59)	.0006	1.25 (1.03–1.51)	.0208	1.06 (0.87–1.30)	.5380
Normal to diabetic (*n* = 64)	28/1204 versus 36/3399	3.58 (2.15–5.97)	<.0001	2.68 (1.47–4.87)	.0013	2.01 (1.07–3.78)	.0291
Stable impaired (*n* = 459)	151/1204 versus 308/3399	1.00 (ref.)		1.00 (ref.)		1.00 (ref.)	
Impaired to diabetic (*n* = 111)	53/1204 versus 58/3399	1.88 (1.23–2.88)	.0037	1.66 (1.02–2.70)	.0426	1.49 (0.88–2.52)	.1395
Impaired to normal (*n* = 86)	20/1204 versus 66//3399	0.64 (0.37–1.10)	.1059	0.78 (0.42–1.44)	.4148	0.82 (0.43–1.55)	.5425
Stable diabetic (*n* = 410)	241/1204 versus 169/3399	1.00 (ref.)		1.00 (ref.)		1.00 (ref.)	
Diabetic to impaired/normal (*n* = 13)	3/1204 versus 10/3399	0.21 (0.06–0.79)	.0203	0.17 (0.04–0.78)	.0225	0.11 (0.02–0.84)	.0334
2‐h glucose							
Stable normal (*n* = 2274)	345/1553 versus 1929/3672	1.00 (ref.)		1.00 (ref.)		1.00 (ref.)	
Normal to impaired (*n* = 935)	248/1553 versus 687/3672	2.10 (1.74–2.53)	<.0001	1.68 (1.36–2.07)	<.0001	1.51 (1.21–1.87)	.0002
Normal to diabetic (*n* = 137)	53/1553 versus 84/3672	3.75 (2.59–5.42)	<.0001	2.67 (1.75–4.08)	<.0001	2.11 (1.34–3.33)	.0012
Stable impaired (*n* = 495)	193/1553 versus 302/3672	1.00 (ref.)		1.00 (ref.)		1.00 (ref.)	
Impaired to diabetic (*n* = 278)	140/1553 versus 138/3672	1.62 (1.20–2.18)	.0017	1.51 (1.07–2.13)	.0180	1.65 (1.14–2.38)	.0074
Impaired to normal (*n* = 301)	93/1553 versus 208/3672	0.68 (0.50–0.92)	.0127	0.76 (0.53–1.07)	.1186	0.81 (0.56–1.17)	.2560
Stable diabetic (*n* = 736)	449/1553 versus 287/3672	1.00 (ref.)		1.00 (ref.)		1.00 (ref.)	
Diabetic to impaired/normal (*n* = 69)	32/1553 versus 37/3672	0.52 (0.32–0.86)	.0115	0.51 (0.29–0.90)	.0205	0.49 (0.26–0.95)	.0336
Insulin resistance							
Stable normal (*n* = 3434)	500/1741 versus 2934/3913	1.00 (ref.)		1.00 (ref.)		1.00 (ref.)	
Normal to resistance (*n* = 813)	317/1741 versus 496/3913	3.90 (3.29–4.64)	<.0001	2.68 (2.22–3.23)	<.0001	2.27 (1.87–2.75)	<.0001
Stable resistance (*n* = 1039)	741/1741 versus 298/3913	1.00 (ref.)		1.00 (ref.)		1.00 (ref.)	
Resistance to normal (*n* = 368)	183/1741 versus 185/3913	0.39 (0.30–0.49)	<.0001	0.51 (0.39–0.67)	<.0001	0.59 (0.44–0.78)	.0002

*Note*: Cutoffs of specific glucose parameters: HbA1c (normal: <5.7%; elevated: 5.7%–6.4%; diabetic: ≥6.5% among diabetes), fasting glucose (normal: <5.6; impaired: 5.6–6.9; diabetic: ≥7.0 mmol/L among diabetes), 2‐h glucose (normal: <7.8 mmol/L; impaired: 7.8–11.0 mmol/L; diabetic: ≥11.1 mmol/L among diabetes), insulin resistance (normal: <2.5; resistance: ≥2.5). The stable levels of glucose parameters tested at both baseline and follow‐up visits, including stable normal, stable impaired/elevated, stable diabetic, and stable resistance were regarded as the reference outcome. Model 1 was adjusted for age, sex, and follow‐up interval; Model 2 was further adjusted for current smoking and drinking (yes or no), education levels (<9 years or ≥9 years), physical activity (<600 MET‐min/week or ≥600 MET‐min/week), body mass index, family history of diabetes (yes or no), hypertension (yes or no); Model 3 was further adjusted for LDL‐cholesterol, HDL‐cholesterol, triglycerides, and HbA1c at baseline in the analysis for fasting, 2‐h glucose, and insulin resistance and further adjusted for fasting glucose in the analysis for HbA1c.

Abbreviations: CI, confidence interval; HbA1c, glycated hemoglobin; HDL, high‐density lipoprotein; LDL, low‐density lipoprotein; MET‐min/week, metabolic equivalent minutes per week; NAFLD, non‐alcoholic fatty liver disease; NGT, normal glucose tolerance; OR, odds ratio.

### 3.4 NAFLD status changes with progression and remission of glycemic metabolism

**FIGURE 3:**
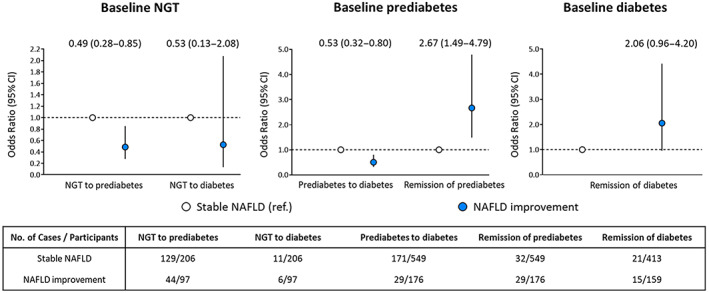
NAFLD improvement with changes of glycemic metabolism. The stable glycemic status at two visits of baseline and follow‐up, including stable NGT, stable prediabetes, and stable diabetes were regarded as the reference outcome. Multivariable models were adjusted for age, sex, follow‐up interval, current smoking and drinking (yes or no), education levels (<9 years or ≥9 years), physical activity (< 600 MET‐min/week or ≥ 600 MET‐min/week), BMI, family history of diabetes (yes or no), hypertension (yes or no), LDL‐cholesterol, HDL‐cholesterol, triglycerides, HOMA‐IR at baseline, and *Δ* BMI. NGT, normal glucose tolerance; BMI, body mass index; CI, confidence interval; HDL, high‐density lipoprotein; HOMA‐IR, homeostasis model assessment of insulin resistance; LDL, low‐density lipoprotein; MET‐min/week, metabolic equivalent minutes per week; NAFLD, nonalcoholic fatty liver disease; OR, odds ratio.

Considering the mutual interaction between NAFLD and glycemic metabolism, NAFLD improvement to some extent mitigated the effect on the progression and remission of glycemic metabolism compared with no NAFLD improvement (Figure 3). In particular, NAFLD improvement decreased the risk of prediabetes progressing to new‐onset diabetes (OR 0.50; 95% CI 0.32–0.80) and increased the probability of prediabetes remission (OR 2.67; 95% CI 1.49–4.79), respectively. Moreover, it had a marginally promoting effect on diabetes remission (OR 2.06; 95% CI 0.96–4.42).

**FIGURE 4:**
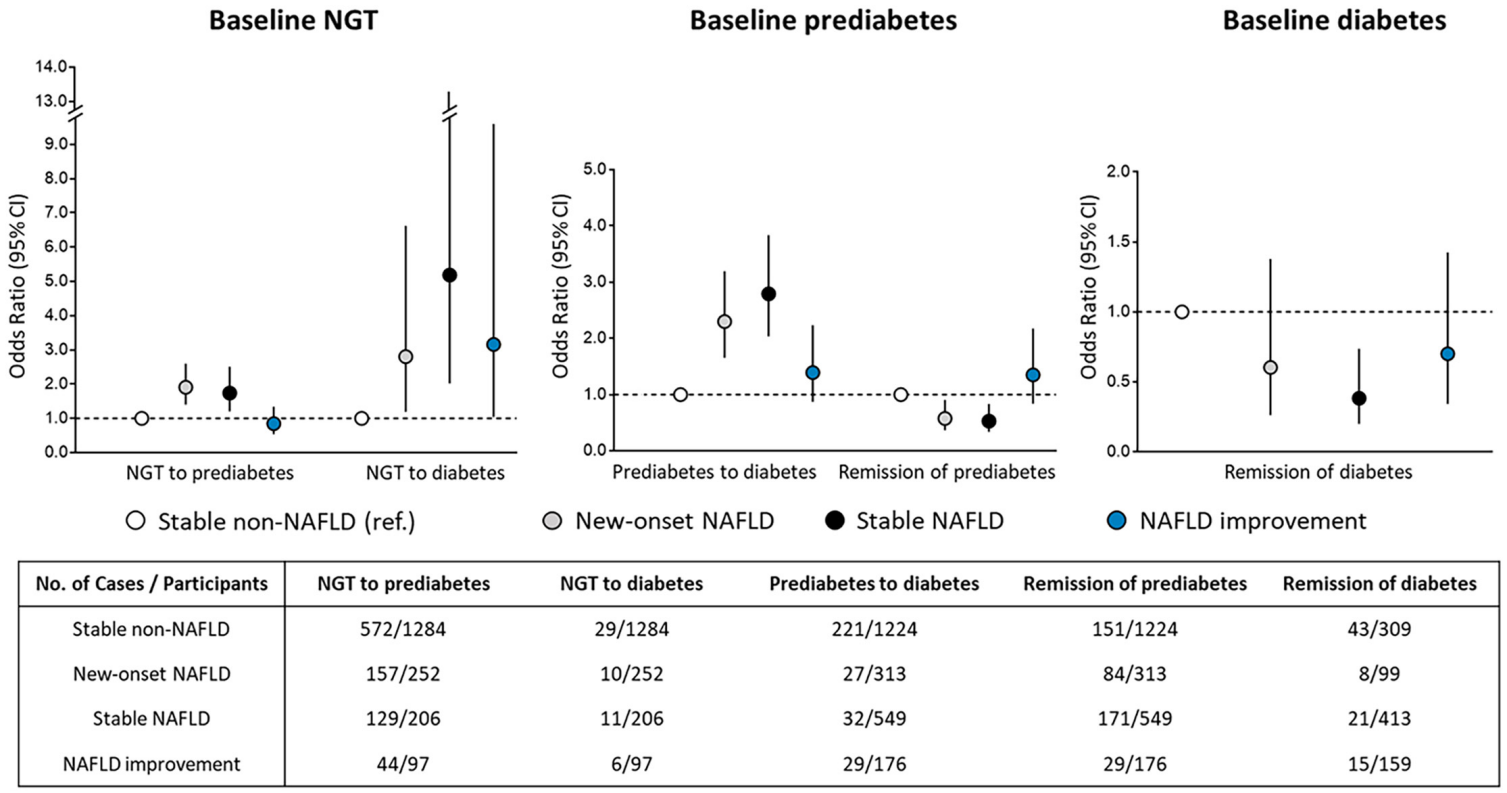
NAFLD status changes with progression and remission of glycemic metabolism. The stable glycemic status at two visits of baseline and follow‐up, including stable NGT, stable prediabetes, and stable diabetes were regarded as the reference outcomes. Multivariable models were adjusted for age, sex, follow‐up interval, current smoking and drinking (yes or no), education levels (<9 years or ≥9 years), physical activity (<600 MET‐min/week or ≥ 600 MET‐min/week), BMI, family history of diabetes (yes or no), hypertension (yes or no), LDL‐cholesterol, HDL‐cholesterol, triglycerides, HOMA‐IR at baseline, and *Δ* BMI. BMI, body mass index; CI, confidence interval; HDL, high‐density lipoprotein; HOMA‐IR, homeostasis model assessment of insulin resistance; LDL, low‐density lipoprotein; MET‐min/week, metabolic equivalent minutes per week; NAFLD, non‐alcoholic fatty liver disease; NGT, normal glucose tolerance; OR, odds ratio.

During the follow‐up period, NAFLD was in a dynamic changing condition. In addition to NAFLD improvement, associations of varying progression of NAFLD, including new‐onset NAFLD and stable NAFLD status with glycemic metabolism should also be considered (Figure 4). Compared with participants who remained stable non‐NAFLD status, NAFLD status changes were associated with glycemic transition at different degrees, of which stable NAFLD tended to show the most significant association with progression of glycemic metabolism, including from NGT to prediabetes (OR 1.74; 95% CI 1.21–2.51) and to diabetes (OR 5.19; 95% CI 2.02–13.29), as well as from baseline prediabetes to diabetes (OR 2.79; 95% CI 2.03–3.83). Meanwhile, stable NAFLD contributed to the least likelihood in remission of prediabetes (OR 0.53; 95% CI 0.34–0.83) and diabetes (OR 0.38; 95% CI 0.20–0.74), respectively.

## 4 DISCUSSION

In this prospective study of community‐based population, we illustrated that the presence of NAFLD was associated with increased risk of glycemic progression and decreased likelihood of remission. Participants with overweight or obesity and higher probability of liver fibrosis tended to aggravate this risk. The association remained consistent with changes of specific glucose parameters, which indicated that NAFLD was involved in the whole process of glucose regulation. In addition, NAFLD status changes simultaneously influenced the transition of glycemic metabolic status, of which NAFLD improvement mitigated the deterioration of glycemic transition whereas NAFLD progression further impeded the likelihood of glycemic remission. Our findings investigated a comprehensive association between NAFLD status and remission and progression along the glycemic continuum, suggesting that diabetes remission and prediabetes normalization were achievable clinical targets among NAFLD population and emphasizing that improvement of liver fat content was conductive to promote glycemic remission.

Recently, the feasibility of diabetes remission has reignited great interest. ADA proposed “remission” as the most appropriate descriptive term and updated the criteria of remission to support future clinical practice.^8^ However, how common the remission of T2D was among the general population remained poorly reported. A cohort investigation from Scotland estimated 4.8% of the prevalence of T2D remission in 2019 among 162 316 national T2D register.^18^ The study further summarized that participants in remission tended to be older, have a lower HbA1c at diagnosis, with no history of antidiabetic prescription, succeed in any weight loss from diagnosis, and have had previous history of bariatric surgery, comparing to participants who did not achieve remission, which were coincided with experience from previous intervention studies.^19^, ^20^ Therefore, ascertainment of those characteristics contributed to the identification of individuals with T2D who were most likely to achieve and maintain remission, which was important for making informed decisions regarding relevant T2D management and intervention.

As NAFLD and T2D have been known to coexist, share common pathogenic factors, and act in synergy to increase the risk of adverse clinical outcomes, assessment of liver fat content was equally essential for the evaluation of T2D remission.^21^, ^22^, ^23^ DiRECT was the first randomized clinical trial to assess the diabetes remission as the primary outcome, which reported 46% of T2D remission at 12 months.^4^ Further discussion regarding the responders who returned to the normal glucose homeostasis was observed in those with profound reduction in liver fat content. The dramatic and sustained normalization of liver fat content was associated with a fall in both plasma triglyceride concentration and intrapancreatic fat content, which contributed to the decreased exposure of β cell to fat metabolites and ultimately β cell function recovery,^7^ suggesting that improving liver fat accumulation was the critical segment to achieving T2D remission.

The association of liver fat accumulation and diabetes remission in population observation study was first reported in Japanese in a 2‐year cohort study.^9^ Among 2567 diabetic participants, presence of fatty liver at baseline was associated with 49% decreased odds of T2D remission. Our findings were in line with the study by assessing a 52% lower probability of T2D remission for NAFLD participants and extended the knowledge to the whole glycemic continuum based on a general population. The results showed that presence of NAFLD not only brought about a decreased likelihood of T2D remission but also 30% lower odds of normalization toward prediabetes. Meanwhile, presence of NAFLD contributed to 236% and 69% higher risks of progression from NGT and prediabetes to T2D, respectively. In addition to the overall transition of glycemic metabolic status, similar tendency was detected in associations of NAFLD and transition of specific glucose parameters, suggesting that great importance should be attached to the evaluation of NAFLD status at very early stage of abnormal glucose metabolism. The earlier NAFLD is identified and improved, the more it can prevent or even reverse the deterioration of glucose metabolism at an earlier stage.

Coinciding with associations of NAFLD status and glycemic metabolic transition, NAFLD participants with overweight or obesity as well as higher probability of fibrosis contributed to an increased risk of glycemic progression and a lower chance of remission, which indicated that the obese status and fibrosis severity tended to be identifiable traits both in the assessment of T2D remission management and NAFLD improvement. On the other hand, there were approximately 10%–20% of individuals with lean NAFLD.^24^ Lean NAFLD represented a distinct phenotype, which was more correlated with metabolic changes, genetic variation, gut microbiota, and skeletal muscle atrophy.^25^, ^26^ Participants with lean NALFD also had metabolic abnormalities but the degree was more favorable than obese NAFLD. Conversely, changes in genetic variation were more prominent in lean NAFLD, such as the GG variant in the patatin‐like phospholipase domain containing 3 (*PNPLA3*) was independently associated with nonalcoholic steatohepatitis and fibrosis.^27^ Discrepancies in pathophysiological mechanisms perhaps accounted for the insignificance of progression from NGT to prediabetes or diabetes and remission of prediabetes or diabetes in lean NAFLD. Therefore, more attention should be focused on hepatic progression in participants with lean NAFLD. Additionally, it was noteworthy that glycemic dysregulation facilitated by NAFLD was independent of BMI change.

With the influence of lifestyle change, NAFLD is simultaneously in a dynamic changing condition. Therefore, NAFLD status changes should be taken into consideration in the transition of glycemic metabolic status. Apart from the improvement of NAFLD, progression of NAFLD was further grouped into new‐onset NAFLD and stable NAFLD. We found that NAFLD improvement was associated with promotion of the remission of T2D and prediabetes, whereas new‐onset NAFLD and stable NAFLD impeded the process of remission to a large extent. In addition, previous studies demonstrated that participants with diabetes and coexistence of hepatic steatosis preoperatively experienced better long‐term glycemic outcomes and predicted a higher chance of T2D remission after gastric bypass surgery, which indirectly reflected the positive effect toward the improvement of hepatic fat.^28^

To the best of our acknowledge, it was the first time to comprehensively assess the associations of NAFLD status changes with progression and remission across a whole spectrum of glycemic continuum. It was highlighted that NAFLD status changes were involved in the whole process of glycemic metabolism. Combined with the mechanism foundation of liver fat accumulation and diabetes remission strengthened by DiRECT and a previous study examining the associations of early abnormal glycemic levels with development and resolution of NAFLD in nondiabetic individuals,^29^ current analysis added a certain weight to the causal relationship of the two metabolic diseases.^21^ Moreover, NAFLD and T2D are often termed comorbidities, driven by the failure to efficiently sequester excess energy, not only sharing interconnected pathogenic factors but also for the synergetic development and progression of end‐organ dysfunction, such as arteriosclerotic cardiovascular disease and progressive chronic kidney disease.^30^ Therefore, our findings stressed the necessity of joint prevention and control on both liver and glucose metabolism.

Several limitations still merited consideration. First, fatty liver was diagnosed by ultrasonography without clarifying the specific liver fat content. However, accumulating evidence has demonstrated that ultrasound‐based screening of fatty liver was applicable in a large‐scale on‐site epidemiological investigation.^31^ Second, data on the duration of diabetes were not accessible so we did not adjust it in models. Because the analysis of glycemic metabolism was not only pointed at diabetes but also transition of prediabetes and normoglycemia, duration of diabetes was not applicable in the latter. Third, skeletal muscle mass is a nonnegligible factor associated with both NAFLD and diabetes remission.^32^, ^33^ Precise assessment of skeletal muscle mass (such as dual energy X‐ray absorption) was lacking but we used the surrogate biomarker of FFM in a sensitivity analysis to improve the robustness.^34^

## 5 CONCLUSIONS

Current novel findings elaborated that presence of NAFLD increased risk of glycemic progression and decreased likelihood of prediabetes or T2D remission. NAFLD improvement mitigated the deterioration of glycemic transition, whereas NAFLD progression impeded the chance of remission to a large extent. Therefore, participants with NAFLD require ongoing support to forestall both progression of hepatic outcome and glycemic metabolism. The results emphasized joint management of NAFLD and T2D and further focused on identifying liver‐specific subgroups of diabetes to tailor and target early intervention to participants who would benefit most.

## FUNDING INFORMATION

The investigators are grateful to all participants for their cooperation in the study. This work was supported by the grants from the National Natural Science Foundation of China (Nos. 81870560, 82070880, 81941017, 81970706, 82022011), Shanghai Municipal Government (No. 18411951800), Shanghai Shenkang Hospital Development Center (Nos. SHDC12019101, SHDC2020CR1001A, SHDC2020CR3064B), the Scientific and Technological Committee of Shanghai (19411964200), Shanghai Jiaotong University School of Medicine (DLY201801), Ruijin Hospital (No. 2018CR002), and Shanghai Rising‐Star Program (21QA1408100).

## CONFLICT OF INTEREST

The authors declare that they have no conflict of interest.

## Supporting information


**Table S1** Sensitivity analysis of FFM‐adjusted association of NAFLD with progression and remission of glycemic metabolism.

## Data Availability

The datasets generated and/or analyzed are available from the corresponding author on reasonable request.
