# Supplementary material for: Nonalcoholic fatty liver disease in relation to the remission and progression along the glycemic continuum
Source: J Diabetes. 2022 Sep 26;14(9):606–19. doi: 10.1111/1753-0407.13314 (PMC9512772; doi:10.1111/1753-0407.13314)
Supplement: Supplementary file 1 — Table S1 Sensitivity analysis of FFM‐adjusted association of NAFLD with progression and remission of glycemic metabolism. [file JDB-14-606-s001.docx]

**Supplementary material**

**Table S1 Sensitivity analysis of FFM-adjusted association of NAFLD with progression and remission of glycemic metabolism.**

|  | **Baseline NAFLD (+)** | | | |
| --- | --- | --- | --- | --- |
|  | **Multivariable adjusted model**  **[OR (95% CI)]** | ***P* value** | **FFM adjusted model**  **[OR (95% CI)]** | ***P* value** |
| **Among participants with NGT (n=2043)** | | | | |
| NGT to prediabetes (n=995) | 1.18 (0.89-1.56) | 0.2531 | 1.11 (0.82-1.50) | 0.4923 |
| NGT to diabetes (n=60) | 3.36 (1.60-7.07) | 0.0014 | 3.24 (1.43-7.32) | 0.0048 |
| **Among participants with prediabetes (n=2501)** | | | | |
| Prediabetes to diabetes (n=479) | 1.69 (1.32-2.17) | < 0.0001 | 1.71 (1.31-2.23) | < 0.0001 |
| Remission of prediabetes (n=351) | 0.70 (0.51-0.98) | 0.0371 | 0.74 (0.52-1.06) | 0.0976 |
| **Among participants with diabetes (n=1127)** | | | | |
| Remission of diabetes (n=102) | 0.48 (0.30-0.78) | 0.0033 | 0.45 (0.27-0.76) | 0.0029 |

Multivariable adjusted model was adjusted for age, sex, follow-up interval, current smoking and drinking (yes or no), education levels (< 9 years or ≥ 9 years), physical activity (< 600 MET-min/week or ≥ 600 MET-min/week), body mass index, family history of diabetes (yes or no), hypertension (yes or no), LDL-cholesterol, HDL-cholesterol and triglycerides at baseline.

Abbreviations: FFM, fat-free mass; NAFLD, non-alcoholic fatty liver disease; OR, odds ratio; CI, confidence interval; NGT, normal glucose tolerance; MET-min/week, metabolic equivalent minutes per week; LDL, low-density lipoprotein; HDL, high-density lipoprotein.
